# Diabetes Distress and Glycemic Control in Type 2 Diabetes: Mediator and Moderator Analysis of a Peer Support Intervention

**DOI:** 10.2196/21400

**Published:** 2021-01-11

**Authors:** Kara Mizokami-Stout, Hwajung Choi, Caroline R Richardson, Gretchen Piatt, Michele Heisler

**Affiliations:** 1 National Clinician Scholars Program Institute for Healthcare Policy and Innovation University of Michigan Ann Arbor, MI United States; 2 Division of Metabolism, Endocrinology and Diabetes University of Michigan Ann Arbor, MI United States; 3 Ann Arbor Veteran Affairs Hospital Ann Arbor, MI United States; 4 Department of Internal Medicine University of Michigan Ann Arbor, MI United States; 5 Department of Health Management and Policy University of Michigan Ann Arbor, MI United States; 6 Department of Family Medicine University of Michigan Ann Arbor, MI United States; 7 Department of Learning Health Sciences University of Michigan Ann Arbor, MI United States; 8 Department of Health Behavior and Health Education University of Michigan Ann Arbor, MI United States

**Keywords:** diabetes mellitus, diabetes distress, health behavior, peer support

## Abstract

**Background:**

High levels of psychosocial distress are correlated with worse glycemic control as measured by glycosylated hemoglobin levels (HbA_1c_). Some interventions specifically targeting diabetes distress have been shown to lead to lower HbA_1c_ values, but the underlying mechanisms mediating this improvement are unknown. In addition, while type 2 diabetes mellitus (T2D) disproportionately affects low-income racial and ethnic minority populations, it is unclear whether interventions targeting distress are differentially effective depending on participants’ baseline characteristics.

**Objective:**

Our objective was to evaluate the mediators and moderators that would inform interventions for improvements in both glycemic control and diabetes distress.

**Methods:**

Our target population included 290 Veterans Affairs patients with T2D enrolled in a comparative effectiveness trial of peer support alone versus technology-enhanced peer support with primary and secondary outcomes including HbA_1c_ and diabetes distress at 6 months. Participants in both arms had significant improvements in both HbA_1c_ and diabetes distress at 6 months, so the arms were pooled for all analyses. Goal setting, perceived competence, intrinsic motivation, and decisional conflict were evaluated as possible mediators of improvements in both diabetes distress and HbA_1c_. Baseline patient characteristics evaluated as potential moderators included age, race, highest level of education attained, employment status, income, health literacy, duration of diabetes, insulin use, baseline HbA_1c_, diabetes-specific social support, and depression.

**Results:**

Among the primarily African American male veterans with T2D, the median age was 63 (SD 10.2) years with a baseline mean HbA_1c_ of 9.1% (SD 1.7%). Improvements in diabetes distress were correlated with improvements in HbA_1c_ in both bivariate and multivariable models adjusted for age, race, health literacy, duration of diabetes, and baseline HbA_1c_. Improved goal setting and perceived competence were found to mediate both the improvements in diabetes distress and in HbA_1c_, together accounting for 20% of the effect of diabetes distress on change in HbA_1c_. Race and insulin use were found to be significant moderators of improvements in diabetes distress and improved HbA_1c_.

**Conclusions:**

Prior studies have demonstrated that some but not all interventions that improve diabetes distress can lead to improved glycemic control. This study found that both improved goal setting and perceived competence over the course of the peer support intervention mediated both improved diabetes distress and improved HbA_1c_. This suggests that future interventions targeting diabetes distress should also incorporate elements to increase goal setting and perceived competence. The intervention effect of improvements in diabetes distress on glycemic control in peer support may be more pronounced among White and insulin-dependent veterans. Additional research is needed to understand how to better target diabetes distress and glycemic control in other vulnerable populations.

## Introduction

Diabetes distress, or the negative emotional and behavioral responses that can occur as a result of having a demanding chronic illness like diabetes, is an increasingly recognized psychosocial factor influencing diabetes self-management [1]. The prevalence of at least moderate levels of diabetes distress is up to 45% in adults with type 2 diabetes (T2D) [2], and high levels of diabetes distress lead to poor medication adherence, higher glycosylated hemoglobin A_1c_ (HbA_1c_) values, and, ultimately, poor quality of life [2-4].

While the link between high levels of diabetes distress and higher HbA_1c_ has been well established [1], a number of evaluated interventions specifically targeting diabetes distress lead to improvements in glycemic control [5]. Examples of such interventions include educational, psychosocial, or psychological programs (including cognitive behavioral therapy, motivational interviewing, and mindfulness-based interventions). Prior RCTs and systematic reviews have elucidated that psychosocial and psychological interventions, particularly those that are tailored specifically for diabetes and have a patient empowerment or motivational interviewing component, are more successful at improving glycemic outcomes in addition to reducing diabetes distress [5-9]. The exact mechanisms behind this relationship are not clear, but drawing on well-established behavioral theories may help to clarify this link. Perceived competence and self-efficacy, or the belief in an individual’s ability to complete a task, is a key feature of social cognitive theory [10], and it has been found to be consistently negatively correlated with distress and is in the mechanistic pathway between diabetes distress and self-management behaviors in T2D [11,12]. It is therefore likely that improving [2] perceived competence is an important element of interventions that improve both diabetes distress and glycemic control. Similarly, self-determination theory postulates that autonomy support, defined as the provision of social support in a way that respects the patient’s values, autonomy, and choice, is an important motivator for patients with chronic disease such as diabetes [13]. As such, autonomy support has also been shown to be an important buffer against the effects of diabetes distress on glycemic outcomes [14]. However, beyond this, there is not a consistent strategic approach common among interventions that improves both diabetes distress and glycemic control. Further elucidation is thus needed to ensure that effective intervention components that improve these constructs are incorporated into future interventions for diabetes mellitus.

Equally important is understanding the characteristics of participants who benefit the most from these interventions. Prior studies have found that patients who are younger, female, have longer duration of diabetes, and are of ethnic minority status, particularly African Americans, have higher diabetes distress levels [15-17]. Interventions targeting specific ethnic minority populations who experience disproportionate diabetes burden and elevated diabetes distress levels have shown mixed findings. These studies, however, are limited by small sample sizes and do not allow comparisons of effects across participants of different ethnicities [18]. Similarly, diabetes-specific characteristics of those who respond to interventions specifically for distress are unknown. As may be anticipated, high diabetes distress levels are associated with fear of insulin use in insulin-naïve patients [19], but it is unclear whether interventions targeting distress are as effective in insulin users as in noninsulin users.

Peer support interventions, in which an individual with prior experience or knowledge who has been successful in their own self-management behaviors serves as a supportive mentor for a target population of patients with similar ethnic or socioeconomic background, are emerging as an important tool for patients with diabetes mellitus, particularly for vulnerable patient populations [14]. Peer support interventions have been successful in improving both glycemic outcomes and psychosocial outcomes, including diabetes distress, and are an attractive, low-cost approach for health care systems [20-22]. A recently published randomized controlled trial (RCT) of peer support versus technology-enhanced peer support for primarily African American veterans with T2D who receive care at an urban Veterans Affairs (VA) health center published by Heisler et al [23] demonstrated that the peer coach model they evaluated, both with and without technology enhancement, was effective at improving glycemic control and reducing diabetes distress over the 6-month intervention period.

In this trial, participants were randomized to peer coaches without any additional eHealth tools or to peer coaches using an individually tailored, web-based educational tool (iDecide) over the course of 6 months. This tool had interactive features to allow participants to understand their personal diabetes risk profile as well as explore options for medications based on cost, effectiveness, and side effects [23]. Peer coaches all received training in motivational interviewing [23]. In this trial, both arms achieved statistically and clinically significant improvements in both diabetes distress and HbA_1c_ without any significant difference between the two intervention arms [23]. This successful trial thus presents an opportunity to explore the psychosocial mechanisms that lead to improvements in glycemic control when diabetes distress is reduced as well as the participant baseline characteristics that may predict responsiveness to such an intervention. The objectives of this study were therefore to evaluate mediators and moderators in the relationship between change in diabetes distress and change in glycemic control over a 6-month period in response to a peer support intervention.

## Methods

### Conceptual Model for Mediator and Moderator Analysis

**Figure 1:**
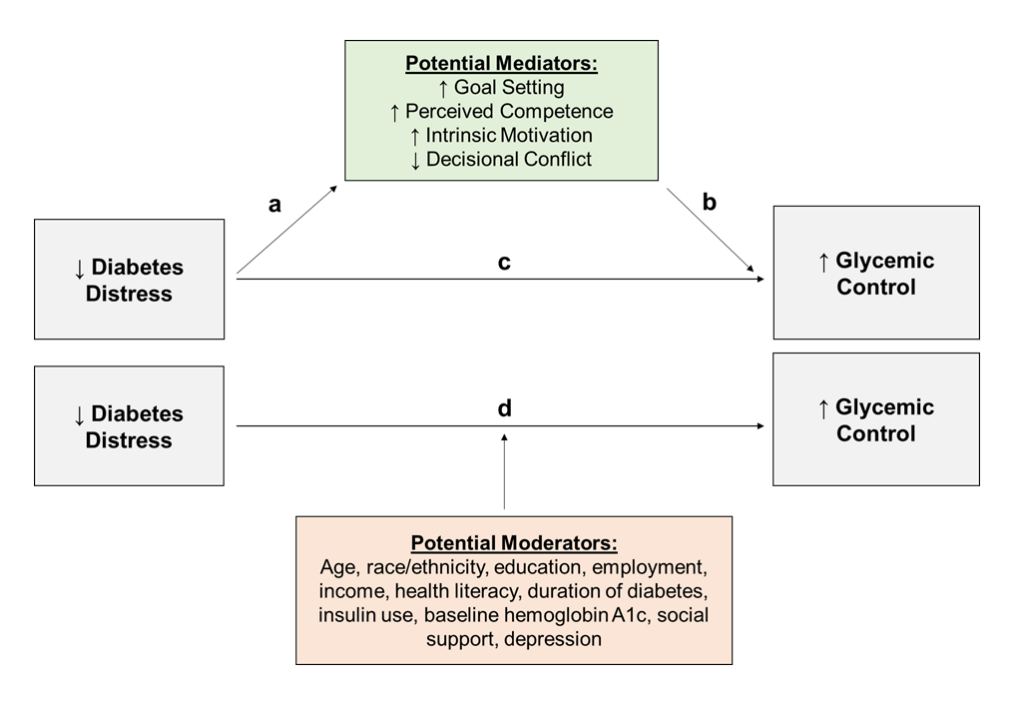
Conceptual model for hypothesized mediators and moderators of improved glycemic control in a peer coaching intervention.

A mediator analysis is one method to explore the psychosocial mechanisms that link diabetes distress and glycemic control. In such an analysis, a conceptual model is created that hypothesizes potential targets, or mediators, along the mechanistic pathway that an intervention must include in order to be successful in achieving the desired outcome. In the previously mentioned RCT by Heisler et al [23], participants had at least weekly contact with a fellow patient with T2D who had received a 2-hour training session with a focus on motivational interviewing, including active listening skills, rolling with resistance, enhancing change talk, goal setting, and action planning. During these sessions, peer coaches helped participants develop and follow up on weekly action steps to meet the participants’ defined behavioral goals. In order to ensure fidelity and help further strengthen the peer coach’s motivational interviewing skills, we held monthly hour-long booster sessions to provide reinforcement and additional training to coaches throughout the intervention period. Based on self-determination theory, which postulates that patients with diabetes who experience more autonomy supportiveness by their health care providers and supporters are more motivated and perceive themselves to be more competent in diabetes self-management, we hypothesized that both intrinsic motivation and perceived competence are important targets in the mechanistic pathway between diabetes distress and glycemic control [24]. Similarly, based on prior studies demonstrating the importance of goal setting and decisional conflict, we hypothesized that both are crucial elements of self-management support interventions to improve both diabetes distress and glycemic control [25]. Our full mediation model is demonstrated in Figure 1 with the pathway through relationship a and relationship b demonstrating the fully mediated model through our hypothesized mediators of goal setting, perceived competence, intrinsic motivation, and decisional conflict.

A moderator analysis can be used to evaluate the characteristics of participants who benefited the most from the peer support intervention of reducing diabetes distress to improve glycemic outcomes. These characteristics are called moderators as they help inform differential effects in the relationship between an independent and dependent variable and hence identify potential modifiers and/or target population for the intervention. In our conceptual model shown in Figure 1, we hypothesized that potential moderators include baseline patient characteristics (age, race, education, employment, and health literacy), certain diabetes characteristics (duration of diabetes, HbA_1c_, and insulin use), diabetes-specific social support, and comorbid depression. Our specific questions were as follows:

- In an intervention that improves both diabetes distress and glycemic control, are improvements in diabetes distress correlated with improvements in HbA_1c_ (main effect)?
- Do goal setting, perceived competence, intrinsic motivation, and decisional conflict work individually or in combination to mediate the relationship between diabetes distress and glycemic control (mediating effect)?
- Does age, race, education, employment, health literacy, duration of diabetes, HbA_1c_, insulin use, diabetes-specific social support, or depression moderate the relationship between diabetes distress and glycemic control (moderating effect)?

### Setting, Recruitment, Intervention, and Measures

The target population for this study included veterans with T2D and high baseline HbA_1c_ values enrolled in a comparative effectiveness RCT of peer support versus technology-enhanced peer support. The description of recruitment, intervention, outcomes, and results of this RCT have been described previously [23]. Glycemic control was measured using HbA_1c_ at baseline and 6 months. Diabetes distress and potential mediators were measured using validated surveys at baseline and 6 months, which were then scaled from 0 to 100, with higher numbers indicating more positive outcomes (eg, lower diabetes distress, higher goal setting). Specifically, the following scales were used (see Multimedia Appendix 1 for further details):

- Diabetes distress: Measured, analyzed, and reported using the 2-item validated Diabetes Distress Scale–2, which assesses feelings that living with diabetes is overwhelming and/or that the participant is failing in their diabetes management [26,27].
- Goal setting: Measured, analyzed, and reported using the 3-item goal setting subscale of the Patient Assessment of Chronic Illness Care, which assesses whether participants were aided in setting goals for self-management and, if so, whether an action plan was developed [28].
- Perceived competence: Measured, analyzed, and reported using the 4-item validated Perceived Competence scale, which assesses the extent to which a participant feels confident and capable of meeting the challenges of diabetes self-management [13].
- Intrinsic motivation: Measured, analyzed, and reported using the intrinsic motivation subscale of the Treatment Self-Regulation Questionnaire, which assesses the extent to which participants feel self-motivated to improve their health behaviors [13].
- Decisional conflict: Measured, analyzed, and reported using the 1-item validated Decisional Conflict Scale, which assess the extent to which a participant is satisfied with their medication options for diabetes [29].

In the RCT, both arms demonstrated improved diabetes distress and HbA_1c_ values at 6 months. Therefore, in this study, participants in both arms were combined to investigate goal setting, perceived competence, intrinsic motivation, and decisional conflict as potential mediators, as shown in Figure 1. Additionally, baseline characteristics were evaluated as moderators of improvement in both diabetes distress and glycemic control, also shown in Figure 1.

### Statistical Analysis

Descriptive statistics were used to evaluate frequencies and means of baseline participant characteristics, and paired *t* tests were used to evaluate the change in means from baseline to 6 months for the independent variable, dependent variable (HbA_1c_), and hypothesized mediator variables (goal setting, perceived competence, intrinsic motivation, and decisional conflict). Bivariate and multivariable linear regressions were used to assess whether the change in diabetes distress at 6 months (independent variable) is associated with the change in HbA_1c_ at 6 months (dependent variable). Covariates include age, race, health literacy, duration of diabetes, and baseline HbA_1c_.

We next assessed the role of goal setting, perceived competence, intrinsic motivation, and decisional conflict as mediators between the change in diabetes distress and the change in HbA_1c_ at 6 months. Multivariable linear regression models were used with the covariate adjustments of age, race, health literacy, duration of diabetes, and baseline HbA_1c_. This is conceptualized by the mediation model in Figure 1:

- Relationship a: between diabetes distress (independent variable) and all potential mediators (dependent variables)
- Relationship b: between all potential mediators (independent variable) and HbA_1c_

The potential mediators that were found to be significantly associated with the change in diabetes distress and HbA_1c_ at 6 months were selected for formal mediation testing by using seemingly unrelated linear regression techniques [30]. We evaluated each individual mediator separately as well as the shared effect of the combined mediators on the mediation pathway through relationships a and b (the indirect pathway) [30]. We calculated bias-corrected 95% confidence intervals from a bootstrapping method with 5000 replications [30].

Finally, sociodemographic factors (age, race, highest attained education, income, employment) and baseline clinical and psychosocial attributes (health literacy, HbA_1c_, duration of diabetes, insulin use, diabetes-specific social support, depressive symptoms) were assessed as potential moderators of the relationship between change in diabetes distress and change in HbA_1c_ at 6 months. Multivariable linear regressions include an interaction term between the change in diabetes distress at 6 months and each of the potential moderators as well as those variables themselves. The change in HbA_1c_ at 6 months was the independent variable in these models and covariates included age, race, health literacy, duration of diabetes, and baseline HbA_1c_ except where the variable was tested as a moderator. This moderator model is conceptualized in Figure 1 (ie, differential effects on relationship d). For each potential moderator, the significance of the interaction term was assessed for different subgroups, and the difference in coefficients between the subgroups was evaluated for significance.

## Results

### Description of the Sample

A total of 290 veterans with T2D were enrolled in the two intervention arms of the RCT. Baseline characteristics of the full cohort are shown in Table 1. Being a veteran population, 98% of the participants were male with an average age of 63 (SD 10.2) years, and 63% were African American. The average HbA_1c_ was 9.1% (SD 1.7%) with a mean of 15 years of diabetes duration, and 60% of the participants were insulin-dependent. At 6 months, diabetes distress improved by 4.8 points (95% CI 2.2 to 7.5; *P*<.001) and mean HbA_1c_ levels improved by 0.7% (95% CI –0.9 to –0.5; *P*<.001) in all participants (Multimedia Appendix 2). Scores for goal setting, perceived competence, intrinsic motivation, and decisional conflict improved by 14.3, 6.9, 6.8, and 6.8 points, respectively (all *P*<.001) at 6 months (Multimedia Appendix 2).

**Table 1:** Baseline characteristics of all participants (n=290).

Characteristic		Value
Age in years, mean (SD)		63 (10.2)
**Gender, n (%)**		
	Female	7 (2)
	Male	283 (98)
**Race, n (%)**		
	Black	181 (62)
	White	106 (37)
	Other	2 (0.7)
**Work status, n (%)**		
	Employed	74 (26)
	Not employed	49 (17)
	Retired	141 (49)
	Disabled	23 (8)
**Education level**		
	Less than high school	12 (4)
	High school graduate	78 (27)
	Some tech or vocational	23 (8)
	Some college or more	177 (61)
**Income ($), n (%)**		
	1-15,000	61 (21)
	16,000-30,000	81 (28)
	31,000-55,000	59 (20)
	56,000 and above	46 (16)
	Prefer not to discuss	42 (15)
Baseline HBA_1c_^a^, mean (SD)		9.1 (1.7)
Number of years with diabetes, mean (SD)		15.2 (10.0)
Insulin use, n (%)		171 (60)
Number of oral antihyperglycemic meds, mean (SD)		1.1 (0.8)
Health literacy, mean (SD)		7.0 (1.9)
Diabetes-specific social support^b^, mean (SD)		54.4 (14.3)
Depression^c^, mean (SD)		76.9 (27.0)

^a^HBA_1c_: hemoglobin A_1c_.

^b^Based on the Diabetes-Specific Social Support Needs assessment [31], scaled score ranging from 0 to 100, with more positive outcomes reflected by higher numbers.

^c^Based on the Patient Health Questionnaire–2 scaled score ranging from 0 to 100, with more positive outcomes reflected by higher numbers.

### Results of the Main Relationship

A significant association between the improvement in diabetes distress and decreased HbA_1c_ was found in the unadjusted model (β-coefficient –0.017; 95% CI –0.028 to –0.006; *P*=.003) (relationship d). This association remained significant in the adjusted model, controlling for age, race, health literacy, duration of diabetes, and baseline HbA_1c_ (β-coefficient –0.015; 95% CI –0.025 to –0.006; *P*=.001).

### Results of the Mediator Analysis

Improvement in goal setting at 6 months was associated with improvements in diabetes distress (β coefficient 0.225, *P*=.02) and reduction in the HbA_1c_ (β coefficient –0.009, *P*=.004) at 6 months. Similarly, improvement in perceived competence at 6 months was associated with both improvements in diabetes distress (β coefficient 0.182, *P*=.002) and the improvement in HbA_1c_ (β coefficient –0.011, *P*=.03) at 6 months. Neither intrinsic motivation or decisional conflict were associated with the change in diabetes distress or change in HbA_1c_ at 6 months so were removed from further mediation analyses. These results are highlighted in Table 2.

**Table 2:** Adjusted estimates of the effect of diabetes distress on all potential mediators (relationship a) and the effect of all mediators on hemoglobin A_1c_ (relationship b).^a^

Potential mediator (outcome in relationship a; predictor in relationship b)	Main predictor: diabetes distress^b^ (relationship a)			Main outcome: hemoglobin A_1c_^c^ (relationship b)		
	β coefficient	95% CI	*P* value	β coefficient	95% CI	*P* value
Goal setting	.225	.036 to .414	.02	–.009	–.015 to .002	.004
Perceived competence	.183	.065 to.300	.002	–.011	–.021 to –.001	.03
Intrinsic motivation	.007	–.127 to.141	.91	–.008	–.017 to .001	.07
Decisional conflict	.101	–.053 to.255	.20	–.007	–.015 to .0003	.06

^a^Diabetes distress, hemoglobin A_1c_, and all potential mediators assessed as the mean change from baseline to 6 months.

^b^Models included diabetes distress as the independent variable and potential mediators as dependent variables; covariates include age, race, health literacy, duration of diabetes, and baseline A_1c_ variables.

^c^Models included potential mediators as the independent variable and hemoglobin A_1c_ as the dependent variable; covariates include age, race, health literacy, duration of diabetes, and baseline A_1c_ variables.

Table 3 presents the extent to which the association between improvement in HbA_1c_ and the improvement in diabetes distress was mediated by goal setting or perceived competence (through the pathway that encompasses relationships a and b in Figure 1). We found that both goal setting and perceived competence are modest mediators with a combined 20% shared total effect (combined indirect effect –0.003, 95% CI –0.0072 to –0.0005).

**Table 3:** Mediating effects of goal setting and perceived competence in the relationship between diabetes distress and hemoglobin A_1c_ (mediator analysis).

Potential mediator^a^	Indirect effect^b^ (95% CI)	Share of total effect (%)
Goal setting	–0.002 (–0.0052 to –0.0001)	13.3
Perceived competence	–0.001 (–0.0045 to –0.0002)	6.7
Combination of goal setting and perceive competence	–0.003 (–0.0072 to –0.0005)	20

^a^Goal setting and perceived competence assessed as the mean change from baseline to 6 months.

^b^Covariates include age, race, health literacy, duration of diabetes, and baseline hemoglobin A_1c_.

### Results of the Moderator Analysis

As shown in Table 4, the within-group estimates for the relationship between the change in diabetes distress and the change in HbA_1c_ at 6 months was significant for participants who are younger than age 65 years, have more than a high school education, are employed, have an income greater than $30,000 per year, have lower health literacy, have more depressive symptoms, who have more social support, who have had diabetes for fewer years, and those with a baseline HbA_1c_ <8.5%. The between group estimates suggest there is a significant difference in the relationship between the change in diabetes distress and the change in HbA_1c_ at 6 months by race and the status of insulin use: stronger for whites compared with African Americans (*P*=.002) and for those who were using insulin compared with those not (*P*=.02).

**Table 4:** Adjusted estimates on the effect of improved diabetes distress on improved glycemic control, by groups with different baseline characteristics (moderator analysis).

Potential moderator		N	Baseline mean diabetes distress (Predictor)	Baseline mean HBA_1c_^a^ (Outcome)	Adjusted estimates			
					β coefficient for change at 6 months (within subgroup)^b^	*P* value	Difference in β coefficients (between subgroups)	*P* value
**Age in years**								
	<65	154	71.7	9.3	–0.019	.002	0.007	.24
	>65	136	74.9	8.8	–0.012	.11		
**Race**								
	Black	181	74.0	9.1	–0.006	.28	0.029	.002
	White	106	72.2	9.0	–0.035	<.001		
**Education**								
	<HS^c^	12	77.8	8.8	0.024	.52	0.040	.63
	>HS	278	73.0	9.1	–0.016	.001		
**Employment**								
	None^d^	213	74.6	9.1	–0.011	.19	0.008	.58
	Employed	74	69.6	8.9	–0.018	.002		
**Income ($)**								
	<30,000	142	73.1	9.1	–0.012	.07	0.011	.13
	>30,000	105	73.8	9.0	–0.023	.003		
**Health literacy**								
	Low	152	70.4	9.1	–0.026	<.001	0.018	.07
	High	138	76.3	9.1	–0.008	.20		
**Baseline depression^e^**								
	Low	132	81.9	8.8	–0.013	.10	0.003	.64
	High	158	66.0	9.3	–0.015	.01		
**Baseline social support^f^**								
	Low	111	76.9	9.2	–0.012	.15	–0.004	.59
	High	130	72.2	9.0	–0.016	.007		
**Duration of diabetes in years**								
	<10	111	71.4	9.3	–0.026	.006	0.016	.05
	>10	179	74.3	8.9	–0.008	.07		
**Baseline HBA_1c_ (%)**								
	<8.5	109	78.1	7.7	–0.021	.004	0.011	.50
	>8.5	134	70.8	10.2	–0.010	.14		
**Insulin use**								
	No	119	73.7	8.8	–0.006	.40	0.024	.02
	Yes	171	72.9	9.3	–0.029	.001		

^a^HBA_1c_: hemoglobin A_1c_.

^b^Adjusted for age, race, health literacy, duration of diabetes and baseline hemoglobin A_1c_ except where these variables were tested as moderators.

^c^HS: high school.

^d^Includes not employed, retired and disabled.

^e^Based on scaled PHQ-2 scores (above and below scaled median value).

^f^Based on scaled DSS scores (above and below scaled median value).

## Discussion

### Principal Findings

We found that in a cohort of primarily African American veterans with T2D, improvements in diabetes distress are associated with improvements in glycemic control as measured by HbA_1c_. Additionally, goal setting and perceived competence are modest mediators of this effect with goal setting and perceived competence accounting for 13% and 7% of the total effect, respectively. Combined, goal setting and perceived competence account for one-fifth of the total shared effect between diabetes distress and glycemic control, suggesting that goal setting and perceived competence are important targets in the mechanistic pathway. Finally, we found that participants with certain sociodemographic and diabetes-specific characteristics are more responsive to improvements in diabetes distress with the peer support approach tested in this RCT. In particular, Caucasian veterans and veterans who require insulin are more likely to demonstrate improved glycemic control with improved diabetes distress. This is an important finding to guide the development of future interventions. Knowing which populations respond to various types of interventions is the first step in personalized care for diabetes self-management to improve both glycemic and psychosocial outcomes.

In this study, we evaluated the results of a peer support RCT for veterans with T2D that demonstrated improvements in both diabetes distress and HbA_1c_ at 6 months to assess for potential underlying mechanisms and baseline participant characteristics that predict both psychosocial and glycemic responsiveness to the intervention. In concert with findings from findings from other studies, we found that diabetes distress is associated with HbA_1c_ [3,32].

Importantly, we also found that perceived competence is a mediator in the pathway between diabetes distress and glycemic control. Although self-efficacy is traditionally associated with the social cognitive theory and perceived competence is an important theme in the self-determination theory, the concepts of self-efficacy and perceived competence are related and often used interchangeably [33]. Multiple studies have demonstrated negative correlations between diabetes distress and self-efficacy, and in one recent study self-efficacy was found to be an important mediator between diabetes distress and glycemic control [2,11]. Our finding that perceived competence is highly associated with both diabetes distress and glycemic control and is in fact in the mechanistic pathway therefore reinforces previous findings.

Our study also had several important novel findings. The first is the importance of goal setting not only as a negative correlate of diabetes distress and glycemic control but also as a mediator in the pathway between diabetes distress and glycemic control. This finding highlights diabetes-specific goal setting as an important target of any intervention to improve both psychosocial and glycemic outcomes. Moreover, we found that certain baseline characteristics predict a more robust improvement of the HbA_1c_ due to the reduced levels of diabetes distress. Race was found to a moderator, suggesting that Caucasian veterans responded more to the peer support intervention than African American patients. Prior studies suggest that peer supporters who are culturally appropriate (including concordant age, race, and gender) may be more effective peer supporters for African Americans with diabetes [34,35]. Given that the burden of T2D falls heavily on minority populations, including African American and Latino populations [36], further studies are needed to understand the characteristics of effective interventions that target these high-risk populations, such as cultural concordance among peer supporters. Additionally, insulin use was found to be a moderator, suggesting that peer support interventions targeting high distress levels in insulin-requiring T2D patients lead to better glycemic control. This is important because approximately one-quarter of T2D patients in the United States currently require insulin, and this proportion is on the rise [37].

### Strengths and Limitations

This study has several strengths. The first is that, to our knowledge, this is the first study looking at mediators and moderators between glycemic control and diabetes distress in an intervention that improves both. We incorporated robust statistical methods to assess the mediation pathway, finding that goal setting and perceived competence are important for future interventions targeting both glycemic and psychosocial outcomes for T2D. This is also one of the first studies to more specifically examine a broad array of socioeconomic and diabetes-specific characteristics that might moderate the relationship between diabetes distress and glycemic control. This is important because this can facilitate screening and targeted interventions using information readily captured by electronic medical records.

We also recognize that our study has several important limitations. First, this study was conducted in primarily African American male veterans with T2D, which limits the generalizability of our findings. It is therefore possible that, in other populations, goal setting and perceived competence have less significance in the mechanistic pathway between elevated levels of diabetes distress and worse glycemic control. Additionally, our use of brief validated scales to measure multiple complicated psychological constructs is a potential limitation, as these short-form scales did not permit in-depth investigation into different facets of these constructs. For example, we used the Diabetes Distress Scale 2 to measure diabetes distress, rather than the full 17-item Diabetes Distress Scale. Although the 2-item Diabetes Distress Scale has been found to correlate well with the larger Diabetes Distress Scale questionnaire, it does not provide subtypes of distress as it only measures emotional distress and this may have impacted our moderator analyses [27]. Prior studies indicate Black patients have higher levels of provider-related distress [38], which was not specifically measured in our study. It is possible that there are differences in the subtypes of diabetes distress (emotional burden, provider-related, interpersonal, and regimen-related) [26] among different populations (such as race/ethnicity) that account for the differential response in White versus Black participants in our study. The study population was also nearly exclusively male and does not therefore generalize to women with T2D, who often have higher levels of diabetes distress [39]. Future studies should include evaluation of interventions of women with T2D with high diabetes distress levels and use of more comprehensive scales to measure diabetes distress in order to more accurately generalize to all T2D populations. Finally, we hypothesized a priori that there would be 4 potential mediators and found that only goal setting and perceived competence were mediators. However, combined, these mediators only accounted for 20% of the mediation effect, suggesting that there are other important mediators in the mechanistic pathway between diabetes distress and glycemic control that we did not measure. Future studies are therefore needed to clarify these additional mediating mechanisms.

### Conclusion

In conclusion, we found that in a peer support intervention for T2D in primarily African American male veterans both goal setting and perceived competence are important mediators in the mechanistic pathway between diabetes distress and glycemic control. Additionally, we found that this peer support intervention that improved diabetes distress was most effective in reducing HbA_1c_ levels in White and insulin-requiring veterans with T2D. These findings are important for informing future interventions that target both psychosocial and glycemic outcomes and efforts to tailor interventions to best meet the needs of patients with different characteristics.

## Appendix

Multimedia Appendix 1 Diabetes distress, goal setting, perceived competence, intrinsic motivation, and decisional conflict scales.

Multimedia Appendix 2 Summary of the change in diabetes distress, change in HbA1c, and hypothesized mediators between baseline and 6 months.

## Abbreviations

HBA_1c_: hemoglobin A_1c_
RCT: randomized controlled trial
T2D: type 2 diabetes
VA: Veterans Affairs
