# Supplementary material for: Diabetes Distress and Glycemic Control in Type 2 Diabetes: Mediator and Moderator Analysis of a Peer Support Intervention
Source: JMIR Diabetes. 2021 Jan 11;6(1):e21400. doi: 10.2196/21400 (PMC7834928; doi:10.2196/21400)
Supplement: Multimedia Appendix 1 [file diabetes_v6i1e21400_app1.docx]

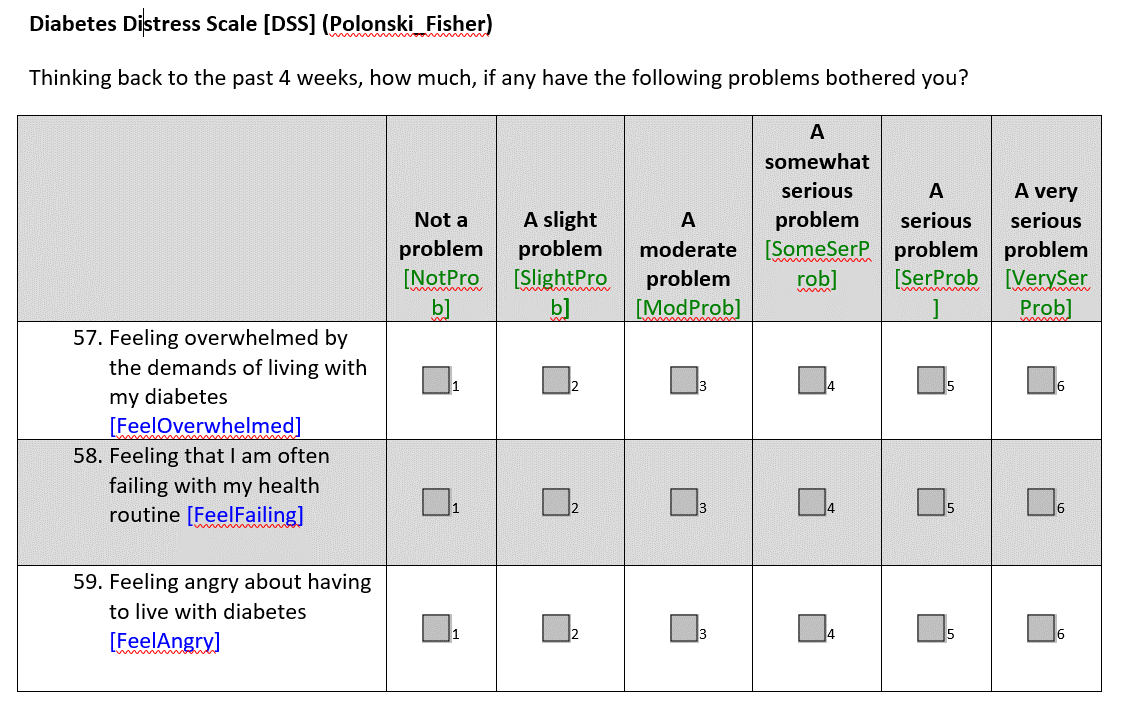


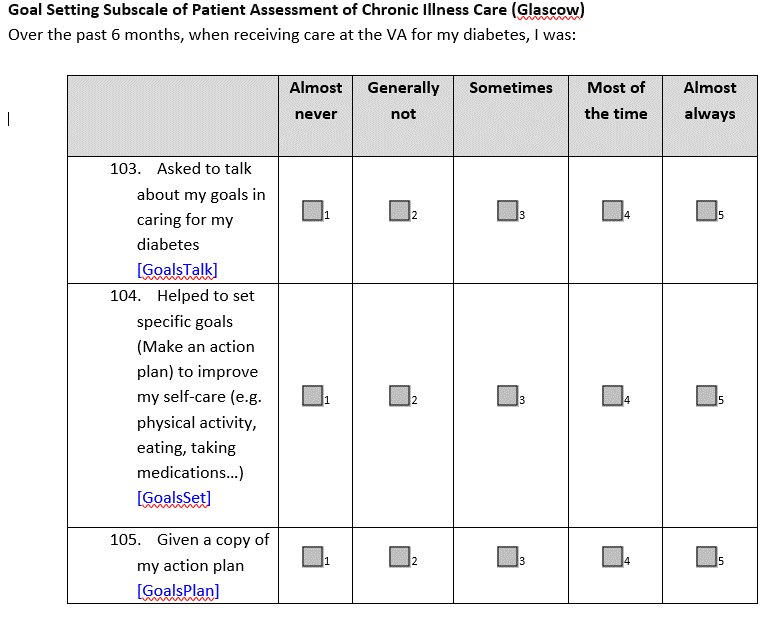


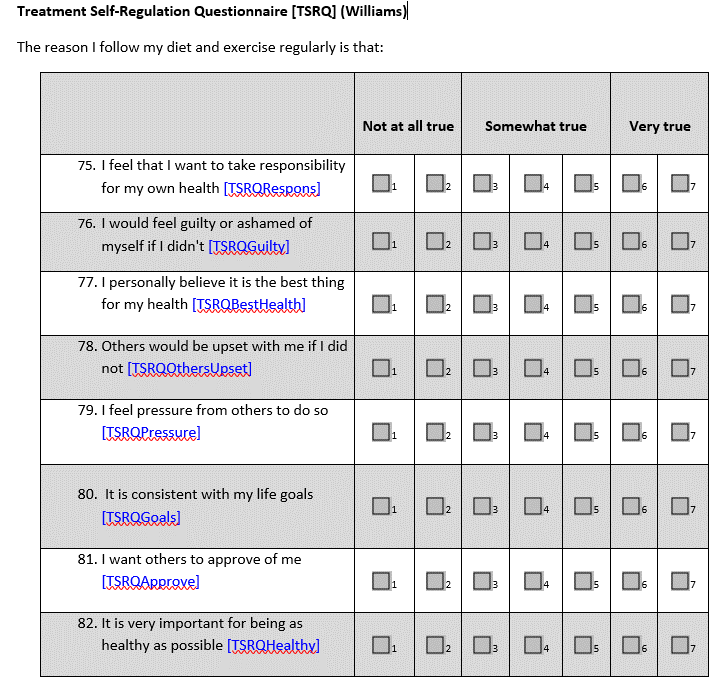


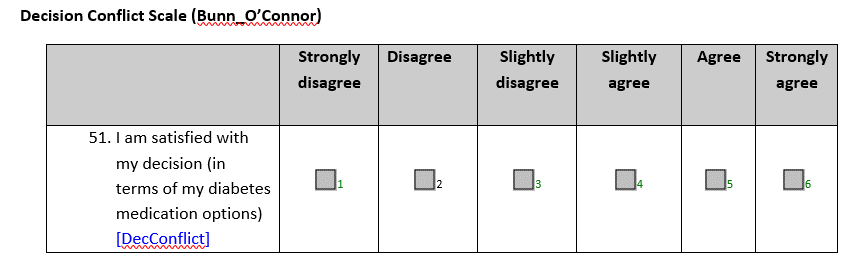


*Questions 75, 77, 80 and 82 comprise the intrinsic motivation subscale of the Treatment Self-Regulation Questionnaire
