# Supplementary material for: Diabetes Distress and Glycemic Control in Type 2 Diabetes: Mediator and Moderator Analysis of a Peer Support Intervention
Source: JMIR Diabetes. 2021 Jan 11;6(1):e21400. doi: 10.2196/21400 (PMC7834928; doi:10.2196/21400)
Supplement: Multimedia Appendix 2 [file diabetes_v6i1e21400_app2.docx]

|  | Outcome measure in time point (unadjusted) | | | | |  | Change over time (adjusted) | | |
| --- | --- | --- | --- | --- | --- | --- | --- | --- | --- |
|  | Baseline | |  | 6 months | |  | From baseline to 6 months | | |
| Variable | *n* | *M* (SD) |  | *n* | *M* (SD) |  | *n* | *M* (95% CI) | *P* value** |
| **Outcome** |  |  |  |  |  |  |  |  |  |
| Hemoglobin A1c | 243 | 9.1 (1.7) |  | 243 | 8.4 (1.7) |  | 243 | -0.7 (-0.9, -0.5) | <.001 |
| **Predictor** |  |  |  |  |  |  |  |  |  |
| Diabetes distress | 242 | 74.3 (23.8) |  | 242 | 79.2 (21.2) |  | 242 | 4.8 (2.2, 7.5) | <.001 |
| **Hypothesized mediators** |  |  |  |  |  |  |  |  |  |
| Goal setting | 241 | 55.5 (28.6) |  | 241 | 69.7 (25.9) |  | 241 | 14.3 (10.4,18.2) | <.001 |
| Perceived competence | 242 | 71.0 (21.0) |  | 242 | 77.9 (19.1) |  | 242 | 6.9 (4.5, 9.4) | <.001 |
| Intrinsic motivation | 241 | 78.2 (21.5) |  | 241 | 85.0 (16.8) |  | 241 | 6.8 (4.1, 9.6) | <.001 |
| Decisional conflict | 242 | 71.7 (25.3) |  | 242 | 78.5 (19.4) |  | 242 | 6.8 (3.6, 9.9) | <.001 |
| *P* values are bolded if <0.05. | | | | | | | | | |
| *All self-reported scales have a range of 0 to 100, with more positive outcomes reflected by higher numbers. | | | | | | | | | |
| ***P* value testing the change from baseline to 6 months | | | | | | | | | |
